# Definitions, phenomenology, diagnosis, and management of the disorders of laughter and crying in amyotrophic lateral sclerosis (ALS): Consensus from ALS and Motor Neuron Disease Scientific Department of the Brazilian Academy of Neurology

**DOI:** 10.1055/s-0043-1771176

**Published:** 2023-08-30

**Authors:** Francisco de Assis Aquino Gondim, Wladimir Bocca Vieira de Rezende Pinto, Marco Antônio Troccoli Chieia, Carolina da Cunha Correia, Francisco Marcos Bezerra Cunha, Mário Emílio Teixeira Dourado Jr, Marcondes Cavalcante França Júnior, Wilson Marques Júnior, Acary Souza Bulle Oliveira, Cleonisio Leite Rodrigues, Delson José da Silva, Elza Dias-Tosta

**Affiliations:** 1 Universidade Federal do Ceará, Departamento de Clínica Médica, Núcleo de Desenvolvimento e Pesquisa de Medicamentos, Fortaleza CE, Brazil.; 2 Universidade Federal de São Paulo, Departamento de Neurologia e Neurocirurgia/Ebserh, Setor de Investigações nas Doenças Neuromusculares, São Paulo SP, Brazil.; 3 Universidade de Pernambuco, Recife PE, Brazil.; 4 Universidade Federal do Cariri, Barbalha CE, Brazil.; 5 Universidade Federal do Rio Grande do Norte, Departamento de Medicina Integrada, Natal RN, Brazil.; 6 Universidade Estadual de Campinas, Departamento de Neurologia, Campinas SP, Brazil.; 7 Universidade de São Paulo, Faculdade de Medicina de Ribeirão Preto, Departamento de Neurociências, Ribeirão Preto SP, Brazil.; 8 Hospital Geral de Fortaleza, Divisão de Neurologia, Fortaleza CE, Brazil.; 9 Universidade Federal de Goiás, Hospital das Clínicas, Unidade de Neurologia e Neurocirurgia/Ebserh, Goiânia GO, Brazil.; 10 Comissão de Ética da Academia Brasileira de Neurologia, São Paulo SP, Brazil.

**Keywords:** Amyotrophic Lateral Sclerosis, Emotional Incontinence, Pathological Laughter and Crying, Pseudobulbar Affect, Affective Symptoms, Emotional Regulation, Affect, esclerose lateral amiotrófica, incontinência emocional, desordens do riso e do choro, afeto pseudobulbar, distúrbios do afeto, regulação emocional, afeto

## Abstract

The spectrum of neuropsychiatric phenomena observed in amyotrophic lateral sclerosis (ALS) is wide and not fully understood. Disorders of laughter and crying stand among the most common manifestations. The aim of this study is to report the results of an educational consensus organized by the Brazilian Academy of Neurology to evaluate the definitions, phenomenology, diagnosis, and management of the disorders of laughter and crying in ALS patients. Twelve members of the Brazilian Academy of Neurology - considered to be experts in the field - were recruited to answer 12 questions about the subject. After exchanging revisions, a first draft was prepared. A face-to-face meeting was held in Fortaleza, Brazil on 9.23.22 to discuss it. The revised version was subsequently emailed to all members of the ALS Scientific Department from the Brazilian Academy of Neurology and the final revised version submitted for publication. The prevalence of pseudobulbar affect/pathological laughter and crying (PBA/PLC) in ALS patients from 15 combined studies and 3906 patients was 27.4% ( *N* = 1070), ranging from 11.4% to 71%. Bulbar onset is a risk factor but there are limited studies evaluating the differences in prevalence among the different motor neuron diseases subtypes, including patients with and without frontotemporal dementia. Antidepressants and a combination of dextromethorphan and quinidine (not available in Brazil) are possible therapeutic options. This group of panelists acknowledge the multiple gaps in the current literature and reinforces the need for further studies.

## Resumo

O espectro de fenômenos neuropsiquiátricos observados na ELA é amplo e não completamente entendido. Desordens do riso e do choro estão entre as manifestações mais comuns. O objetivo deste estudo é relatar os resultados de um Consenso organizado pela Academia Brasileira de Neurologia para avaliar definições, fenomenologia, diagnóstico, e manejo dos distúrbios do riso e do choro em pacientes com ELA. Doze membros da Academia Brasileira de Neurologia – considerados experts na área – foram recrutados para responder 12 questões na temática. Depois da verificação das revisões, um primeiro manuscrito foi preparado. Após, foi realizado um encontro presencial em Fortaleza, Brasil, em 23/09/2022, para discussão do conteúdo. A versão revisada foi posteriormente enviada por e-mail para todos os membros do Departamento Científico de DNM/ELA da Academia Brasileira de Neurologia e a versão final revisada foi submetida para publicação. A prevalência da síndrome pseudobulbar em pacientes com ELA em 15 estudos combinados com 3906 pacientes foi de 27,4% ( *n* = 1070), variando entre 11,4% e 71%. Início bulbar é um fator de risco, mas há limitados estudos avaliando as diferenças em prevalência entre os diferentes subtipos de Doença do Neurônio Motor, incluindo pacientes com e sem Demência Frontotemporal. Antidepressivos e uma combinação de dextrometorfana e quinidina (indisponíveis no Brasil) são opções terapêuticas possíveis. Esse grupo de panelistas reconhece as múltiplas demandas não atendidas na literatura atual e reforça a necessidade de futuros estudos.

## INTRODUCTION

A wide variety of neuropsychiatric disorders have been recognized in amyotrophic lateral sclerosis (ALS) patients with and without dementia. 1 Language, executive dysfunction, and the disorders of laughter and crying (DLC) are the most prevalent disturbances described. 2

The DLC are characterized by complex phenomenology and still not fully understood. They are frequent in several neurological conditions, especially after stroke and ALS. 3 Feelings are considered as mental experiences that accompany body states, while emotions are action programs triggered by external stimuli, complicated patterns of chemical-neural responses responsible for maintenance of life. 4 In ALS, pseudobulbar affect is traditionally considered to be a disorder of emotional expression, although recent evidence also demonstrates abnormal feeling processing. 5 6 However, several authors use the term emotion for both the subjective and objective responses. 7 8 Herein, we will present a consensus about DLC in ALS patients.

## METHODS

A group of 12 Brazilian neurologists, members of the ALS/MND Scientific Department of the Brazilian Academy of Neurology and considered to be representative experts on the subject, was formed to discuss the definitions, phenomenology, diagnosis, and management of the disorders of laughter and crying in ALS patients. The consensus attempted to serve as an educational tool for neurologists from the Brazilian Academy of Neurology.

On 4/2022, an invitation letter was sent to the 12 panelists. After acceptance, the coordinator emailed 12 questions and assigned one specific question to each panelist. Each participant also received a list of papers generated by a PubMed search with the key words ALS, Motor Neuron Disease (MND), Emotional Incontinence (EI), Involuntary Emotional Expression Disorder (IEED), Pseudobulbar Affect (PBA), Emotional Lability, and Pathological Laughter and Crying (PLC), comprising a total of 366 papers (search conducted on 5/16/2022). Each panelist was allowed to consult additional common web-based search engines. After one month, the consensus coordinator collected and reviewed the initial texts written by each participant and proposed corrections, reviewed by each participant. A first draft was emailed and after revision by all participants, a second draft was written and emailed to each participant. On 9/23/2022, during the Brazilian Congress of Neurology, the 12 panelists got together in the city of Fortaleza to discuss the controversial points and vote for the final set of the recommendations. A final draft was sent for review to all members of the Scientific Department of ALS/MND of the Brazilian Academy of Neurology. One month later, the panelists held an online meeting and voted for the final text.

## RESULTS

### What is the spectrum of neuropsychiatric phenomena in ALS patients?

Although considered to be primarily a disease of the motor system, cognitive and behavioral changes were reported in ALS patients after Charcot's landmark description, in the last part of the XIX century. 9 The association between ALS and frontal lobe dysfunction was credited to Von Braunmuhl, 1932. 1 10

ALS is currently recognized as a multisystem disorder. The spectrum of neuropsychiatric changes in ALS is wide and varies according to disease subtypes, reaching the maximum degree of abnormality in the overlap with Frontotemporal Dementia (FTD). In 2017, the ALS Society of Canada sponsored an international consensus to revise the Strong diagnostic criteria for frontotemporal dysfunction in ALS. 11 The consensus highlighted the heterogenous phenomenology affecting over half of ALS patients, expanding the idea of a frontotemporal spectrum disorder of ALS (ALS-FTSD) to: pure motor ALS, ALS with FTD, behavior or cognitive dysfunction not sufficient to meet dementia diagnosis but sufficient to be detected (ALS behavior impairment- ALSbi or ALS cognitive impairment- ALSci), and a small amount of ALS dementia not typical of FTD (ALS dementia). The following neuropsychological domains are affected in ALS patients:

- Executive and social cognition dysfunction: impairment of verbal and letter fluency, difficulty in reasoning, coordinating rules, mental heuristics, abnormal emotional processing, reduced capacity to recognize facial expressions and understanding social situations;
- Language dysfunction: difficulties in word retrieval, sentence processing, spoken and pragmatic language;
- Memory impairment: isolated memory deficits do not meet the criteria for ALSci. Memory deficits are variable and a guide range of deficits has been reported, most delayed verbal memory;
- Behavioral/neuropsychiatric symptoms: apathy is the most common behavior symptom, affecting up to 70% of ALS patients. Disinhibition, egocentric behavior, perseverative and stereotyped behavior, and change of dietary habits are less common than apathy.

Lastly, the disturbances of emotional expression are a significant hallmark of ALS dysfunction and will be the central focus of the present consensus.

Registry-based research has detected a high rate of psychiatric disease in ALS. 12 Depression, neurotic disorders, schizophrenia, history of drug abuse or dependence were associated with increased risk for ALS. 12 Depression may affect 27–41% of the ALS patients. 12 Anxiety and adjustment disorders are also common (⅓), 12 including increased rates of suicide especially in the early stages in some populations. 13

### What are the different terminologies, definitions and subtypes of the DLC?

Laughter and crying are considered abnormal when inappropriate, uncontrollable and/or continuous. 14 The DLC attracted the curiosity of physicians, scientists, and philosophers since ancient times. 15 16 Charles Darwin pointed out that “certain brain diseases, such as hemiplegia, brain loss, and senile decay, have a special tendency to induce crying” 16 . During the late XIX and XX centuries, several terms were used to describe abnormalities of laughter and crying. Epileptic episodes associated with laughter are labeled as gelastic seizures (from the Greek *gelos* , referring to a Greek god or daimon, personification of laughter). More rarely, seizure episodes associated with crying are called dacrystic seizures (from the Greek word *dakryon* for tear). Laughter marking the onset of an apoplectic event, usually a stroke, was first described in 1903 17 and labeled as “ *fou rire prodromique* ” (prodrome of crazy laughter) while acute crying episodes in the setting of strokes “ *folles larmes prodromiques* ” (crazy prodrome of weeping).

For patients with a chronic disorder, such as ALS, the simple facilitation of laughter and crying is known as: EI, pathological emotionality, emotionalism, “ *Affektinkontinenz* .” They all refer to enhancement of laughter and crying, with variable degree of usage by different authors for the description of associated experiential aspects of the emotion itself. *Rires et pleurs spasmodiques* (spasmodic laughter and crying) was one of the oldest terms employed to describe laughter and crying deprived of emotion. Wilson proposed the classic term PLC with the same meaning as *rires et pleurs* spasmodiques. 18

Oppenheim is credited for first using the term PBA 19 to highlight the motor alterations of pseudobulbar palsy (bilateral corticobulbar tract impairment). However, the term PBA can be misleading. 5 20 Recent evidence suggests that corticobulbar tract dysfunction alone is neither necessary nor sufficient to cause PLC. 21 Cummings and other authors have proposed the term IEED to replace PBA and PLC. 8 The belief is that IEED is less pejorative for patients, containing a description of phenomenology and avoiding conceptual confusion between affect and mood.

### What are the clinical features of the DLC in ALS patients?

The disturbances of emotional expression and processing are common in ALS patients. ALS patients may be more likely to laugh or cry either spontaneously or after minor emotional stimuli. This exaggerated emotional expression pattern is classically known as EI. It has been observed in several neurological disorders and reported to be common in stroke patients as early as in 1895 by Edouard Brissaud. 15 S.K. Wilson was one of the first to also report that patients with ALS also laugh or cry under stimulation with a stimulus of opposite valence, e.g., cry after experiencing a happy experience or laugh under sad circumstances (PLC). 18 In addition to this pattern of abnormalities, evidence from laboratory evaluations has documented that patients with ALS also exhibit abnormal processing of emotional stimuli. 5 6 Contrary to prior views, episodes of uncontrollable laughter and crying were most frequently associated with experience of emotional distress, supporting the idea that they were associated with enhanced activation of all channels of emotional response (emotional hyperactivity due to dysfunction of neural systems that control voluntary regulation of emotion). In most cases, the triggers for episodes of laughter and crying were the same as the thoughts and stimuli that could induce crying and laughter in anyone, but they led to rapidly developing high intensity, uncontrollable bursts. 5 6 Laughter episodes had a greater tendency to occur without an obvious precipitant. Of note, considering the classic distinction between PLC and EI, most episodes described by Olney are consistent with EI rather than true PLC. 5 In addition, the phenomenology can be mixed and suddenly evolve from an exaggerated emotional response to a truly opposite response to a trigger, e.g., laughter after a sad stimulus. Following a laboratory paradigm, Hübers et al. 2016 were able to demonstrate altered ability to judge emotional content in ALS patients with PLC. 6 Although uncontrolled laughter and crying episodes were previously thought to be devoid of personal emotional experience (feelings), this is probably not the case. The abnormal pattern of facial movements can be uncomfortable or even painful and associated with spasms, thus receiving the jargon of spasmodic laughter or crying. 15

### What is the prevalence/epidemiology of DLC in ALS patients?

DLC are common in several neurological diseases, including ALS, Parkinson's disease (PD), multiple system atrophy-cerebellar type, multiple sclerosis (MS), stroke, traumatic brain injury (TBI), and Alzheimer's disease (AD). 22 Depending on population, diagnostic criteria, and methodologies, the prevalence of the DLC vary considerably across different neurological conditions, such as in ALS where it occurs most commonly at moderate to advanced disease stages and earlier in individuals with bulbar-dominant or upper motor neuron-dominant compromise. Work et al. determined that the prevalence of PBA in a group of patients with AD, ALS, MS, stroke, TBI and PD ranged from 9.4% to 37.5% depending upon the scale and the threshold used. 23 In a recently published meta-analysis study, ALS patients showed a PBA prevalence of 38.5%, which is higher than other neurodegenerative diseases. 22

We selected cross-sectional, case-control, cohort and experimental studies that had a random sampling, standard criteria for diagnoses of PBA and described the point prevalence of PBA/PLC. The characteristics of included studies were detailed in Table 1 . The prevalence of PBA/PLC in ALS patients from 15 combined studies and 3906 patients was 27.4% ( *N* = 1070), ranging from 11.4% to 71%. The studies demonstrated that PBA/PLC was not uncommon in ALS. Prospective studies, using population-based ALS cohorts, are required for addressing knowledge gaps using trustworthy diagnostics for PLC/PBA.

**Table 1:** Pseudobulbar affect (PBA)/Pathological laughter and crying (PLC) prevalence estimates in patients with ALS

Study, year	Country	Study design	Diagnostic scale (cutoff score if applicable)	Number of subjects	Prevalence (%)
Zigler, 1930 26	USA	Retrospective	NR	101	18.8
Gallagher et al. 1989 27	USA	Cross- sectional	Clinical assessment only NR	73 Younger than 45	49.3
Palmieri et al. 2009 28	Italy	Case-control	ELQ	41	71
Work et al. 2011 23	USA	Online survey	CNS-LS ≥ 13 PLACS ≥ 13 CNS-LS ≥ 21	225	50 32.5 27.5
Brooks et al. 2013 29	USA	Cross- sectional	CNS-LS ≥ 13	125	44.8 PBA
Floeter et al. 2014 30	USA	Retrospective	NR	37 ALS 50 PLS	32.4 ALS 62 in PLS
Tortelli et al. 2016 31	Italy	Cohort	CNS-LS ≥ 13	132	34.09
Thakore et al. 2017 32	USA	Cohort	CNS-LS ≥ 13	735	28.4
Christidi et al. 2017 33	Greece	Case-control	CNS-LS ≥ 13	56	50
Patel et al. 2018 34	USA	Cross- sectional	CNS-LS ≥ 13	26	34.6
Tortelli et al. 2018 20	Italy	Cohort (population-based)	CNS-LS ≥ 13	94	36
Barc et al. 2020 35	Europe, multicenter	Cross- sectional, multicenter	Direct neurology questioning	1145	17.38
Tu S et al. 2021 36	Australia	Retrospective	CNS-LS ≥ 13	35	54.29
Wei QQ et al. 2021 37	China	Cohort	Face-to-face interviews Neurologist	1031	11.4
Chowdhury et al. 2021 38	India	Cross- sectional	CNS-LS≥ 13	50	68

Abbreviations: ALS, amyotrophic lateral sclerosis; CNS-LS, Center for Neurologic Study-Lability Scale; ELQ, Emotional Lability Questionnaire; NR, Not Reported; PLACS, Pathological Laughing and Crying Scale; PLS, primary lateral sclerosis.

### Are there different classifications for the DLC?

Traditionally, the DLC have been classified by Poeck according to the appropriateness of the emotional expression in relation to the emotional trigger, hence grossly divided into 2 subgroups: PLC and EI. 14

In PLC, exaggerated emotional responses occurs after exposure to neutral stimuli or stimuli with opposite emotional valence (e.g., sad stimulus triggering laughter). In EI, episodes are facilitated but happened in the appropriate context, e.g., easy crying in sad situation and easy laughter in happy contexts. Laboratory studies reported that outbursts of PLC may occur under emotionally appropriate conditions. 5 6 In this regard, IEED encompassed both PLC and EI, as it emphasizes the expressive aspect of the emotion. 8 Lauterbach and colleagues provided a criterion-based classification for IEED, splitting the disorder again into PLC, EI and a third subtype with PBA and dysarthric bulbar speech, dysphagia or disinhibited facial and gag reflexes. 24

Gondim and colleagues proposed a new classification for the DLC based on mechanisms, phenomenology (including associated phenomena) and following the premises of the somatic marker hypothesis. 25 This classification attempted to unify all neuropsychiatric disorders leading to altered laughter and crying. Subtypes include motor (emotional expression pathways), sensory (feeling processing) and mixed disorders, further subdivided into positive or negative depending on the mechanism involved. ALS patients would exhibit 3 subtypes: negative motor, sensory or mixed. In this classification, most ALS patients are more prone to negative motor forms. 25 To date, there is no agreement on either the terminology or classification for the DLC.

### What are the possible mechanisms underlying the DLC in neurological disorders and particularly in ALS patients?

Historically, the first group of theories (peripheral), wrongly attributed distinct impairment of different facial nerve fibers. 15 18 In 1879, Nothnagel proposed the existence of a “psychoreflex pathway” to explain the different forms of facial movement paralysis, e.g., voluntary versus emotional facial movements. 18 In 1887, Bechtereff proposed that the expressive centers for facial movements were located in the anterior thalamus and that abnormal laughter resulted from impaired voluntary control and exaggerated involuntary stimuli. 15 18 Wilson proposed the existence of a supranuclear control center for synkinesis of facial and breathing movements linking the VII and X brainstem nuclei and phrenic nerves. PLC was the result of imperfect control of the voluntary paths from the motor cortical areas with the concomitant activation of synkinetic faciorespiratory pathways, e.g., corticofugal pathways to the faciorespiratory centers in the pons and medulla independent of the voluntary cortico-ponto-bulbar tracts to the same nuclei. 18 On excitation they would either arrest or accelerate and interfere with the normal rhythmic activity of the respiratory center. Autopsy and neuroimaging lesion analysis expanded the knowledge about structures involved in acute (e.g., *fou rire prodromique* ) and chronic DLC. Lesions were reported in brainstem, cerebral hemispheres, and subcortical-diencephalic circuits. 25 Recently, a review of previous reports involving lesion-symptom correlation in PLC detailed 70 distinct focal lesions. 39 Klingbeil and colleagues proposed a two-hit model for PLC, e.g., a combination of direct lesion and indirect diaschisis effects cause PLC through loss of inhibitory cortical control of a dysfunctional emotional system. 39 Two PLC subnetwork systems were proposed: a positive and a negative. However, this model is biased for the evaluation of the DLC due to focal brain lesions, such as after strokes. For neurodegenerative diseases, this model is more difficult to be employed. In ALS-associated PLC, functional and structural neuroimaging studies also supported the role of subcortico-thalamo-ponto-cerebellar network pathways in its pathophysiology, and no correlation with sensory deafferentation. 2 27 40 41 42 43

Volitional facial movements are generally preserved in patients with emotional facial paresis, due to distinct excitatory pathways from the frontal and temporal cortices and hypothalamus to the periaqueductal gray matter and inhibitory modulation pathways from the lateral premotor cortices areas. 39 Similar pathway dysfunction has been observed in ALS patients secondary to the reduction or lack of inhibitory circuits originating from the frontal cortex due to progressive loss of neurons from the cortical areas (“ *top-down theory* ”). 5 6 As pointed out by Olney et al. 2011 and others, 5 6 there is substantial evidence of altered feeling processing in ALS patients. In addition to possible involvement of serotonergic pathways, frontal lobe dysfunction may cause impairment of the mirror-neuron network, thus explaining why a primarily motor disease would cause altered feeling processing. More recent studies also emphasized other neurobehavioral aspects of ALS, such as alexithymia. There is marked reduction of gray matter volume of prefrontal cortices, parahippocampal gyri, and right superior temporal pole in patients with ALS. 44

In non-demented patients with ALS and PLC, multimodal neuroimaging studies have disclosed reduced volume of the left orbitofrontal cortex gray matter, putamen, frontal operculum, and bilateral frontal poles. There were also white matter abnormalities involving associative fibers and ponto-cerebellar tracts. 33 Lesions in the medial inferior frontal area were linked to PLC in MS. 21 PLC in ALS has been also evaluated by diffusion-tensor brain MRI imaging and disclosed decreased fractional anisotropy in structures of the cortico-ponto-cerebellar pathways. 30 The higher frequency of PLC among patients with bulbar-onset ALS and the involvement of brainstem motor nuclei correlated with reduced gray matter volume of the brainstem, especially in patients with prefrontal executive dysfunction. 45 Thus, basal ganglia and cortico-ponto-cerebellar pathway involvement may be due to diaschisis from a primary frontal lobe disease.

### What are the available screening tools for the diagnosis and severity quantification of DLC?

The diagnosis of DLC is usually made primarily on clinical grounds, e.g., behavioral evaluation based on clinical history and neurological exam performed by an experienced physician. As detailed on the epidemiology section, the prevalence of those disorders solely based on clinical evaluation may yield false low sensitivity, especially in earlier disease stages. 37 Therefore, several instruments for the diagnosis and quantification of the DLC have been designed. Although more frequently tested in stroke patients, they are also good for the evaluation of all types of DLC, including ALS. None of these instruments have been translated and/or validated into Portuguese.

The first designed tool for the evaluation of PLC was developed in 1993 46 and is known as pathological laughing and crying scale (PLACS). This instrument was an interviewer administered rating scale designed to document the benefit of the treatment with nortriptyline on 82 ischemic stroke patients. PLACS rates sixteen items, scores the severity of each symptom on a 0–3 point scale generating a final sum score. PLC is distinguished by a score of 13 or higher. The interrater reliability was 0.93 with excellent test-retest reliability (0.85) at 2-week intervals.

The Center for Neurologic Study-Lability Scale (CNS-LS) was the first self-reported measure, validated to evaluate affective lability in a large population of ALS patients. 47 In contrast to PLACS, it has been used as an important endpoint in several clinical trials for ALS patients. It consists in a short, self and easily administered questionnaire of 7 items with two subscales: one for laughter (4 items) and one for tearfulness (3 items). The CNS-LS quantifies perceived aspects of PBA, including frequency, intensity, lability, degree of voluntary control, and inappropriateness to context. For each item, respondents indicate on a five-point scale (where 1 = applies never and 5 = applies most of the time) how often they experience symptoms of PBA. A score of 13 on the CNS-LS, as on the PLACS, distinguishes patients with PLC, predicting neurologist's diagnosis, with sensitivity of 0.84 and specificity of 0.81. The total CNS-LS scores showed test-retest reliability of 0.88 for ALS patients. The scale focus on the burden of subjective symptoms over 2 weeks prior to assessment, but provides an accurate indicator of episode frequency. 48

After detecting several problems with PLACS, e.g., reliance on self-rating, insufficient period of assessment, inadequate exploration of appropriateness of emotion, the “emotional lability questionnaire” (ELQ) was validated in ALS patients as modified version of PLACS. 49 ELQ evaluate symptoms experienced by the patients up to 4 weeks prior to screening, which helps to capture patients who experience less frequent episodes. 29 Each questionnaire contains 33 items, divided into 11 questions for laughter, 11 for crying and a specific section on abnormal smiling with 11 questions. ELQ has 2 components, one self-rated and the other given to caregivers. Answers are given in a 4-point Likert scale. One of the ELQ strengths is the inclusion of caregiver's perspective. In ALS, there is good agreement between patient and caregiver scores, confirming patient's symptom awareness. 49 Reports are substantially different in ALS-FTD patients. 50

### What is the differential diagnosis and adequate work-up for the evaluation of the DLC in ALS patients?

When one evaluates a patient with abnormal or exaggerated laughter and crying, the first step is to establish whether there is a mood or a primary disorder of the laughter and crying expression. Mood is an emotional state sustained over long periods (days-weeks or more). A patient with depression feels depressed most of the time and crying is the primary reflection of the depressed mood. 51 The maniac state of bipolar disorder has been linked to exaggerated laughter with euphoria (the so-called maniac laughter). Depression and mania can be diagnosed with a structured anamnesis, however, for the quantification of depression and anxiety there are multiple inventories such as the Hamilton depression rating scale and Beck Depression Inventory. It is also well known that illicit drugs (e.g., marijuana) and substances like Nitrous Oxide (NO) and intravenous valproic acid can induce disordered laughter. 15 Obviously, patients with ALS can also be affected by co-morbidities such as strokes and other structural lesions (e.g., tumors) leading to PLC/PBA. 24 25 39 Proper neuroimaging testing could easily differentiate those conditions.

Therefore, the next step will be to sort out whether there is concomitant PLC/PBA and depression or just one of those two disorders. PLC/PBA is frequently underdiagnosed or misdiagnosed as a mood disorder, especially when there is predominant crying. PBA with depression has more explosive crying episodes, with shorter duration, and no longstanding internal sadness. Depressed individuals possess a persistent mood of sadness, but they do not tend to have frequent crying episodes and, if they happen, they last much longer than PBA. Other symptoms observed in depression are generally not observed in PLC/PBA. 52

There are good screening tools to quantify PLC/PBA: CNS-LS and PLACs. If there are additional symptoms suggestive of dementia, a more thorough neuropsychological battery testing is advisable to establish whether there is restricted or more widespread evidence of cognitive impairment. The use of the Edinburgh Cognitive and Behavioral ALS Screen (ECAS) is an important assessment to detect general aspects of cognitive and behavioral changes in patients with ALS. The Frontal Assessment Battery (FAB) or the ALS Cognitive Behavioral Screen (ALS-CBS) may be used to assess the frontal lobe executive function. 53 54 FAB score <16 or ALS-CBS <10 may define executive dysfunction. 53 54 The global cognition function may be evaluated by the Montreal Cognitive Assessment (MoCA) and the traditional Folstein Mini-Mental State examination.

### Are there any differences in the epidemiology of the DLC in ALS patients based on ALS clinical subtypes?

Considering all the common neurodegenerative disorders, ALS is the leading cause of PBA/PLC. 22 The presence of PBA may further aggravate the disability in affected subjects, as it may worsen social interactions and impair quality of life. 38 Two independent studies found PBA to be more prevalent in depressed patients with ALS compared with non-depressed patients. 32 55 It has been also related to prefrontal cognitive dysfunction. 45 Most reports identified an association between PBA and bulbar onset. 31 32 One of the largest studies was published by Thakore & Pioro. 32 They assessed a database of 735 patients with available self-reported cognitive/behavioral information and found PBA in 28.4% of all subjects. They noticed a significant association with female gender, bulbar onset and presence of upper motor neuron signs: 10%, 29% and 39% for LMN-predominant disease, typical ALS and UMN-predominant disease, respectively. 32 Only 8% of patients with PMA had PBA compared with 40% of PLS patients. Patients with bulbar onset symptomatology appear disproportionately affected. 28 36 37 Few reports failed to replicate this association and found PBA to be as frequent in spinal versus bulbar-onset ALS. 38

### Are there known genetic mutations explaining the different subtypes of neuropsychiatric phenomena in ALS patients?

New genetic data highlighted the well-known overlap between MND and FTD. Considering the fast and major ongoing developments, the reader is referred to latest reviews for each candidate gene involved in ALS/FTD. 56

Multisystem disorder with possible cognitive involvement in MND led to the idea of MND-FTD continuum. 9 57 The spectrum of degenerative MND may encompass a pure motor neuron involvement without cognitive compromise. Genetic forms linked to restricted MND without cognitive involvement include most forms of *SOD1, ANG, VAPB and OPTN* variants. 57 In a recent Brazilian study with 70 *C9orf72* negative patients, and including 7 patients with *VAPB* variants, 23% of the patients were diagnosed with ALSbi and apathy followed by dysphoria and anxiety were the most prevalent findings in the ALSbi subgroup. 58 Chiò and colleagues evaluated 2839 ALS patients in Italy and disclosed important associations between ALS phenotype versus age, sex and genetics. 59 *C9orf72* expansions correlated with bulbar phenotype and were less frequent in pure upper motor neuron forms. *SOD1* variants correlated with flail leg phenotype and were less frequent in bulbar-onset. ALS-FTD correlated with *C9orf72* and bulbar phenotypes. Although *SOD1* is commonly not linked to neuropsychiatric disease, *SOD1* p.Ile113Thr variant can develop cognitive impairment. 60 *FUS* gene mutations may occasionally be associated with mild cognitive impairment. 59

Pathological hexanucleotide repeat expansion in the *C9orf72* gene is the most common monogenic cause of ALS (39–45%) and ALS-FTD spectrum. 61 In most cases, expansions are linked to the FTD behavior variant. Occasionally, it is also associated with the nonfluent/agrammatic or semantic variants of Primary Progressive Aphasia (PPA). 62 Among the psychiatric symptoms, the most relevant are apathy, social isolation, delusion and working memory impairment. 61 *TBK1* gene is associated with ALS-FTD and PLS-dementia. 63 They may develop behavior changes, non-fluent aphasia, memory impairment. FTD may be the presenting manifestation. Other genes associated with the overlap of MND-FTD include *SQSTM1, CHMP2B, CCNF, and TIA1* . Patients with *SQSTM1* variants usually develop the behavior variant FTD, including aggressiveness, mood changes, social detachment, speech apraxia and visuo-constructional deficits. 64 Variants in the *CHCHD10* gene represents up to 3% of ALS-FTD and are frequently associated with complex phenotypes, including ALS-FTD, myopathy and cerebellar ataxia. 65 *VCP* gene variants are associated with ALS cognitive impairment, FTD, myopathy and Paget disease of bone. 66 There are no specific genetic subtypes linked to PBA or PLC in ALS patients.

### Are there phenomenological differences (for DLC) between ALS patients with and without FTD and with complex neuropathological involvement?

ALS is a multisystem disorder. Although primarily a disease of the pyramidal neurons and pathways, there is evidence to support direct cerebellar, basal ganglia, autonomic and even distal axonal (small fiber) involvement or at least due to secondary effects of impaired pyramidal connections (diaschisis) on different systems. Furthermore, it is becoming evident that neuropsychiatric involvement is variable and phenotypically diverse. 67

Cognitive impairment in ALS ranges from no discernible deficit to severe dementia, depending on disease stage and subtype. FTD may precede or follow the onset of motor symptoms in ALS. There are multiple reports suggesting that PLC/PBA is more prevalent in patients with bulbar-onset and predominant upper motor neuron involvement. However, there is only one study that compared neuropsychiatric phenomena, including PBA in pure ALS, behavioral variant of FTD (bvFTD) and ALS-FTD. 9 Among 250 participants (115 with ALS, 98 bvFTD, 37 ALS-FTD) a similar pattern of neuropsychiatric symptoms and symptom severity was observed among the 3 groups. In this study, disinhibition was predominantly related to *C9orf72* hexanucleotide repeat expansion. Indirect evidence points toward lower prevalence of laughter in bvFTD/right temporal variant FTD than ALS since patients with bvFTD and right temporal variant FTD laugh less across both contexts of self and partner speech than healthy controls, bvFTD laugh less relative to their own speech in comparison with controls. 68 In the non-fluent variant of PPA group, laughter was increased in the partner context in comparison to healthy controls. 68 In summary, current evidence do not enable us to conclude that PLC is more prevalent in ALS patients with or without FTD versus pure FTD and further studies are necessary to address this matter.

Lastly, due to the low prevalence of the multiple and rare FTD variants and multisystem neurodegenerative disorders with different neuropathological substrates (TDP43, tauopathy, synucleinopathies), it is not possible to sort out the differences in PLC/PBA prevalence in conditions like three-in-one syndrome (PPA, corticobasal degeneration and frontal lobe dementia), 69 *C9orf72* with combined Multiple System Atrophy and ALS, 70 especially within different disease stages.

### How are those conditions treated? What are the different types of medications available for the treatment of the DLC in ALS patients?

PLC is frequently treated in the context of patient with other clinical comorbidities and potential drug interactions and adverse events. Most clinicians consider the existence of other neuropsychiatric changes during the decision process of the best therapeutic option, including therapies for associated dementia, cognitive decline, mood disorders, and anxiety. 71 Since PLC is more frequently observed in patients with bulbar-onset ALS, upper motor neuron-predominant ALS phenotypes and ALS with distinct cognitive dysfunction, clinicians generally consider other signs, symptoms and complications present in these scenarios during management decisions. 32 72 73 The therapeutic range necessary to partially improve PLC symptoms is generally lower than the daily doses used for the treatment of mood disorders. 74

Although classically used by most neurologists in clinical practice, selective serotonin reuptake inhibitors (SSRI) and tricyclic anti-depressant (TCA) have been only rarely evaluated by specific studies for the treatment of PLC in MND/ALS, and no double-blinded placebo-controlled clinical trials were performed so far to evaluate MND/ALS-associated PLC with antidepressants. 2 Current clinical practices result from previous case series and placebo-controlled trials developed for the treatment of PLC associated with other neurological conditions. These studies included SSRIs (sertraline 50 mg/day, fluoxetine 20 mg/day, citalopram 20 mg/day, escitalopram 10 mg/day), TCAs (nortriptyline 25 mg/day, amitriptyline up to 100 mg/day), dual serotonin-norepinephrine reuptake inhibitors (duloxetine, venlafaxine), mirtazapine, valproate, dopamine agonists (levodopa, amantadine), and non-competitive N-methyl-D-aspartate receptor antagonists (memantine and dextromethorphan-quinidine/DMQ). 2 71 74 75 76 77 78

DMQ targets both N-methyl-D-aspartate receptor as a non-competitive glutamate antagonist and a sigmar-1 receptor agonist. 48 The first FDA-approval of a specific drug for PLC treatment was made in 2010, with the association of dextromethorphan hydrobromide with quinidine sulfate (Nuedexta ^®^ , Avanir Pharmaceuticals), which provided a reduction of almost 50% of emotional lability episodes (crying or laughing). 72 79 In 2004, a randomized trial was published and evaluated DMQ combination (30/30 mg, twice daily), versus dextromethorpan and quinidine alone groups in patients with ALS and PLC. 80 There was marked improvement in the frequency and intensity of PLC episodes and in the quality of life of patients using the DMQ combination. During the first weeks of treatment, almost one quarter of patients withdrew from treatment due to several mild to moderate side effects, 48 80 such as diarrhea, nausea, cough, somnolence, flu-like symptoms, and dizziness. 74 81 The frequency and severity of such adverse events may be markedly reduced by a slow introduction of the therapy, reaching the target dose with two pills a day after 1 week of treatment. 71 81 DMQ association results in more sustained plasma therapeutic levels of dextromethorphan due to quinidine potential to inhibit cytochrome P450 2D6. In 2010, a similar study using a group with lower quinidine dose 10 mg led to lower rates of adverse events in the 30/10 mg and 20/10 mg, BID, and marked improvement of the frequency of PLC episodes. 78 In some patients, DMQ could lower bulbar symptoms (dysphagia and dysarthria) after 12 weeks of follow-up. 78 There are no clinical trials comparing efficacy of DMQ and other anti-depressant drugs in PLC. 74 81 Combined DMQ drugs are still unavailable in Brazil and have not been evaluated or approved by the Brazilian Health Regulatory Agency (ANVISA). The main drugs used for PLC treatment are summarized on Table 2 .

**Table 2:** Level of evidence and grades of recommendation of current available treatments for PLC, according to current evidence-based Medicine tools

**Grades of recommendation**		
Grade A: systematic review of randomized controlled trials (Level 1a); individual randomized controlled trials (Level 1b) Grade B: systematic review of cohort studies (Level 2a); cohort study or low-quality randomized control study (Level 2b); systematic review of case-control studies (Level 3a); case-control studies (Level 3b) Grade C: low-quality cohort, case-control or case series (Level 4) Grade D: expert opinion with non-systematic reviews of results (Level 5)		
**Drug class**	**Posology (Usual dosage)**	**Grade of recommendation**
*Selective Serotonin Reuptake Inhibitors (SSRI)*		
Citalopram	5–20 mg per day	Grade C (Level 4)
Escitalopram	5–20 mg per day	Grade C (Level 4)
Fluoxetine	20–60 mg per day	Grade C (Level 4)
Fluvoxamine	50–300 mg per day	Grade C (Level 4)
Sertraline	50–200 mg per day	Grade C (Level 4)
Paroxetine	20–50 mg per day	Grade C (Level 4)
*Tricyclic Antidepressant (TCA)*		
Amitriptyline	10–100 mg per day	Grade B (Level 2b)
Nortriptyline	10–100 mg per day	Grade B (Level 2b)
Imipramine	10–20 mg per day	Grade C (Level 4)
*Serotonin-norepinephrine reuptake inhibitors*		
Duloxetine	40–60 mg per day	Grade C (Level 4)
Venlafaxine	37.5–225 mg per day (BID)	Grade C (Level 4)
*Dopamine-norepinephrine reuptake inhibitors* Bupropion	150–300 mg per day	Grade C (Level 4)
*Norepinephrine-serotonin modulator* Mirtazapine	15–45 mg per day	Grade C (Level 4)
Dextromethorphan/Quinidine 20 mg/10 mg	1 pill per day, for 7 days; then adjust to 1 pill, BID	Grade A (Level 1b)
*Other drug classes*		
Amantadine	5–200 mg, BID	Grade C (Level 4)
Carbidopa/levodopa	10/100mg or 25/250mg, BID-QID	Grade C (Level 4)
Lamotrigine	50–150 mg per day	Grade C (Level 4)

Abbreviations: BID, two times a day; QID, four times a day; TID, three times a day.

In conclusion, the spectrum of neuropsychiatric phenomena in ALS is wide and not fully understood. DLC stand among the most common manifestations. PBA/PLC affects between 11.4–71% of ALS patients. Bulbar-onset is a risk factor, but there are no adequate studies evaluating the prevalence among different MND subtypes, including patients with and without FTD. Antidepressants and a combination of dextromethorphan and quinidine (not available in Brazil) are possible therapeutic options. This group of panelists acknowledge the multiple gaps in the present literature and the need for further studies.
